# Comparative Risk of Acute Anemia and Urinary Tract Infection With Sulfamethoxazole-Trimethoprim Versus Dapsone After Kidney Transplant

**DOI:** 10.1177/10600280251367427

**Published:** 2025-12-04

**Authors:** Artemis R. Huntsman, Christian J. Squires, Joy Dray Vongspanich, Jeremiah J. Duby

**Affiliations:** 1 Solid Organ Transplant Specialty Pharmacy, University of California Davis Medical Center, Sacramento, CA, USA; 2 Primary Care, University of California Davis Medical Center, Sacramento, CA, USA; 3 Infectious Disease and Solid Organ Transplant, University of California Davis Medical Center, Sacramento, CA, USA; 4 Research and Outcomes, University of California Davis Medical Center, Sacramento, CA, USA; 5 University of California San Francisco School of Pharmacy, Sacramento, CA, USA

**Keywords:** dapsone, SMX-TMP, anemia, kidney transplant, UTI

## Abstract

**Background:**

Dapsone is an alternative prophylactic agent for opportunistic infection in kidney transplant recipients (KTRs) with sufficient glucose-6-phosphate dehydrogenase levels. Potential disadvantages include increased risk for anemia and urinary tract infection (UTI) with dapsone.

**Objective:**

The objective of this study was to compare the risks of anemia and UTI in KTRs who received prophylaxis for opportunistic infection after renal transplant with dapsone versus sulfamethoxazole-trimethoprim (SMX-TMP).

**Methods:**

This single-center, retrospective, cohort review was conducted at a large academic health system. Adult patients who received SMX-TMP or dapsone prophylaxis after renal transplant were included. The primary outcome was risk of acute anemia within 90 days of transplant. Secondary outcomes included the risk of UTI post-transplant. Log-rank test was used for time-to-event analysis for the primary outcome.

**Results:**

A total of 153 patients were included in the analysis. Patients were more likely to experience acute anemia while on dapsone compared with SMX-TMP (hazard ratio = 2.13, 95% confidence interval = 1.04-4.38, *P* = 0.04). No differences were observed in the rates of blood transfusion (14.5% vs 18.4%, *P* = 0.52) or use of erythropoiesis-stimulating agents (40.2% vs 48.7%, *P* = 0.331) between SMX-TMP and dapsone groups, respectively. The prevalence of UTI was comparable between groups (23.4% vs 23.7%, *P* > 0.999).

**Conclusion and Relevance:**

The risk of anemia was twofold higher in KTRs who received dapsone compared with SMX-TMP but did not appear to affect the rates of erythropoiesis-stimulating agent use or blood transfusion. There was no difference in the risk of UTI. These findings may inform agent selection and monitoring of prophylaxis for opportunistic infection in KTRs.

## Background

Kidney transplant recipients (KTRs) are at risk for opportunistic infections in the post-transplant period due to immunosuppressive therapy required to prevent allograft rejection. Sulfamethoxazole-trimethoprim (SMX-TMP) is considered a first-line agent for prophylaxis of common pathogens (eg, *Pneumocystis jirovecii, Toxoplasma gondii*) and urinary tract infections (UTI).^1^ Dapsone is an alternative agent for patients who cannot tolerate SMX-TMP due to sulfa allergy, hyperkalemia, or other adverse effects; however, there are potential disadvantages of dapsone therapy.

Foremost, anemia is a known adverse reaction of dapsone use. The hydroxylamine derivatives of dapsone are associated with oxidative stress that may manifest as hemolysis. Glucose-6-phosphate-dehydrogenase (G6PD) deficiency amplifies the risk and impact of this phenomenon as it compromises hepatic capacity to regenerate the cytoprotective antioxidant glutathione.^2^ Insidious, acute anemia, may compound chronic anemia due to prolonged bone marrow suppression associated with end-stage renal disease. Moreover, anemia-related reductions in oxygen delivery and transfusion-induced immune sensitization may elevate the risk of allograft rejection.^3,4^ In addition, dapsone offers negligible UTI prophylaxis in a population with notable risk factors (eg, ureteral stents, immunosuppression).

Dapsone-related anemia has been previously described in patients with human immunodeficiency virus (HIV) and in transplant recipients.^5-8^ However, the available reports are relatively limited in scope and generalizability. Furthermore, the effects of full-dose prophylaxis (ie, 100 mg daily) with dapsone have not been well-described in patients after renal transplant. The objective of this study was to compare the risks of anemia and UTI in KTRs who received prophylaxis for opportunistic infection after renal transplant with dapsone versus SMX-TMP.

## Materials and Methods

This single-center, retrospective cohort study was conducted in the ambulatory care setting at a large academic health center. This study was approved by the institutional review board. A waiver of informed consent was granted due to the retrospective design and minimal risk posed to participants. Adult patients who received a renal transplant (1/1/2017 to 12/31/2020) and SMX-TMP or dapsone for prophylaxis were eligible for inclusion. Patients were excluded who did not have adequate follow up (<3 months) or were deceased at time of chart review.

### Transplant Protocol

All patients received anti-thymocyte globulin (ATG) with weight-based and risk-based dosing (2-6 mg/kg) as a part of induction therapy. Patients were started on antibiotic prophylaxis on postoperative day 4 with SMX-TMP 400/80 mg or dapsone 100 mg by mouth daily. Sulfamethoxazole-trimethoprim doses were adjusted for renal function based on estimated clearance creatinine. Antibiotic prophylaxis was continued for up to 90 days at the discretion of the transplant team. Sulfamethoxazole-trimethoprim was the first-line agent for prophylaxis of opportunistic infection; patients with allergy or intolerance to SMX-TMP were initiated on or transitioned to dapsone. Patients were cohorted into the SMX-TMP and dapsone groups based on the prophylactic agent at the end of therapy. Maintenance immunosuppression regimens included tacrolimus and mycophenolate with or without prednisone. The standard target range for tacrolimus was 8 to 12 ng/mL for the first 3 months after transplant. Ureteral stents were typically removed by approximately 6 weeks post-transplant. Erythropoiesis-stimulating agents (ESAs) were considered for KTRs whose hemoglobin remained below 10 g/dL. Blood transfusions were generally reserved for patients with hemoglobin levels less than 7 g/dL. All patients who received dapsone were tested for G6PD deficiency prior to initiation. Glucose-6-phosphate-dehydrogenase deficiency was defined as a level less than 9.9 units/g; the maximum measurable level was reported as 21 units/g.

### Study Outcomes

Data were collected and validated through review of the electronic medical record by the study investigators. The primary outcome of this study was the risk of acute anemia after starting antibiotic prophylaxis (ie, postop day 4) and within 90 days of transplant. Acute anemia was defined as a hemoglobin (Hgb) drop of 2 g/dL from baseline, postop levels.^6^ Baseline Hgb was defined as the Hgb value on the day that antibiotic prophylaxis was initiated. If a patient switched from SMX-TMP to dapsone, then the Hgb value at the time of dapsone initiation was used as the baseline Hgb. Secondary outcomes included the time to onset of UTI, severity of anemia, and serum creatinine (SCr). Urinary tract infection was defined by positive urine culture within 90 days of renal transplant. Episodes of UTI and acute anemia were attributed to the prophylaxis that the patient was receiving at the time the event occurred. The time to event was determined from the start of the associated antibiotic therapy. For example, if a patient switched from SMX-TMP to dapsone, then any subsequent UTI was credited to the dapsone cohort; the time to event was determined from the start date of the dapsone.

### Statistical Analysis

A sample of 136 subjects (ie, 68 in each group) was necessary to achieve 90% power to detect a 15% difference (ie, 25% vs 10%) in the primary outcome.^4^ Proportions were compared using χ^2^ and Fisher exact tests, as appropriate. Continuous variables were analyzed using Student *t* test for normally distributed data (eg, means) and Mann-Whitney *U* test for non-normally distributed data (eg, medians). The log-rank test was used to compare distributions for time-to-event analysis and estimate crude hazard ratios (cHR) and 95% confidence intervals (95% CI). Adjusted hazards ratios (aHR) were derived using Cox proportional hazards model and potential covariates for anemia (ie, antibiotic prophylaxis, baseline Hgb, baseline SCr, donor type) and UTI (ie, antibiotic prophylaxis, ureteral stent duration, sex, proximity to induction therapy). Spearman rank correlation analysis was performed to determine the strength and direction of the relationship between G6PD level and change in Hgb level in patients who received dapsone. A significance level of 0.05 was used for all tests.

## Results

**Figure 1.**
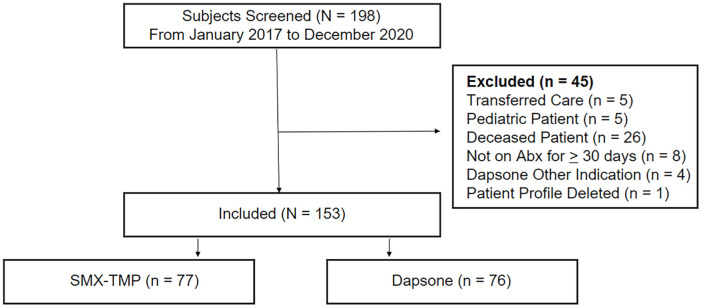
CONSORT diagram illustrating how patients were screened.

A total of 198 KTRs were eligible for inclusion during the study period (Figure 1). 153 patients were included in the final analysis; patients were evenly divided between SMX-TMP (n = 77) and dapsone (n = 76) groups. Baseline demographics were comparable (Table 1). Most KTRs were men (63.4%) and a plurality self-reported Hispanic ethnicity (39.2%). Most KTRs (78.4%) received a deceased donor kidney and experienced immediate graft function (72.5%). There were differences in baseline SCr and baseline Hgb at the time of antibiotic initiation (Table 1). There was no significant difference in maintenance immunosuppression between groups (Table 1). Most patients received 750 mg of mycophenolate mofetil (ie, equivalent to 540 mg mycophenolate sodium) twice daily.

**Table 1.** Kidney Transplant Recipients’ Baseline Characteristics (n = 153).

Characteristic	Total (n = 153)	SMX-TMP (n = 77)	Dapsone (n = 76)	Difference^a^ (95% CI)	*P* value
Age, years (mean ± SD)	47.9 ± 13.9	48 ± 14.2	47.8 ± 13.6	0.2 (-4.21 to 4.61)	0.944
Female sex, n (%)	56 (36.6%)	33 (42.9%)	23 (30.3%)	12.6 (2.5 to 27.7)	0.107
Race: n (%)					
Other	60 (39.2%)	33 (42.9%)	27 (35.5%)		
White	52 (34.0%)	20 (26.0%)	32 (42.1%)		
Asian	26 (17.0%)	17 (22.1%)	9 (11.8%)		
Black	15 (9.8%)	7 (9.1%)	8 (10.5%)		
Weight, mean (kg)	78.7 ± 16.6	75.1 ± 16.9	82.4 ± 15.7	-7.3 (-12.5 to -2.1)	0.007
Deceased Donor, n (%)	120 (78%)	57 (74%)	63 (83%)	-9 (21.9 to 3.93)	0.18
Dialysis Needed on POD4+, n (%)	42 (27%)	20 (26%)	22 (29%)	-3 (-17.1 to 11.1)	0.68
Baseline Hgb mean (g/dL)	10.0 ± 1.6	9.7 ± 1.5	10.3 ± 1.6	-0.6 (-1.09 to -0.11)	0.016
Baseline SCr mean (mg/dL)	4.1 ± 3.5	4.85 ± 0.67	3.29 ± 0.93	1.5 (0.17 to 2.83)	0.007
G6PD Level (U/g) Mean +/- SD	-	-	13.95 ± 2.69	-	-
Mycophenolate dose, n (%)					
1000mg/720mg	1 (<1%)	0 (0%)	1 (1%)		
750mg/540mg	134 (88%)	70 (91%)	64 (84%)	1.56	0.45
500mg/360mg	17 (11%)	7 (9%)	10 (13%)	(0.60 to 4.06)^b^	
250mg/180mg	1 (<1%)	0 (0%)	1 (1%)		
Dapsone Indication, n (%)					
Allergy			27 (36%)		
Hyperkalemia			41 (54%)		
Other			8 (11%)		

Abbreviations: SD, standard deviation; POD4+, postoperative day 4; G6PD, glucose-6 phosphate dehydrogenase; CI, confidence interval.

a Difference of means or percentages (95% CI).

b Odds ratio of mycophenolate dose >500 mg.

Kidney transplant recipients who changed from SMX-TMP to dapsone transitioned at a median of 16 (interquartile range [IQR] = 1-27) days from renal transplantation; hyperkalemia was the most common reason for switching therapy (Table 1). The median duration of prophylaxis was 87 (79.5-96.5) days for the SMX-TMP group compared with dapsone therapy was 69 (IQR = 49-87.75) days (Table 2).

**Table 2.** Acute Anemia and UTI Outcomes.

	Overall (=153)	SMX-TMP (n=77)	Dapsone (n=76)	P-value
Prophylaxis Duration (days), median (IQR)	84 (62.5 – 90)	87 (79.5 - 96.5)	69 (49 - 87.8)	< 0.0001
Acute anemia, n (%)	30 (19.6)	10 (13.0)	20 (26.3)	0.0433
Nadir Hgb (g/dL), mean	8.95 ± 1.7	8.87 ± 1.5	9.03 ± 1.9	0.565
Nadir Hgb From Transplant (days), median (IQR)	21 (5.5 – 42.5)	6 (4 – 19)	39 (23 – 63.3)	< 0.001
Nadir Hgb From Antibiotic Initiation (days), median (IQR)	11 (0 - 23)	1 (0 – 15)	19 (7.25 - 32.8)	< 0.0001
Decrease in Hgb from Baseline (g/dL), mean	1.18 ± 1.23	0.82 ± 0.89	1.28 ± 1.4	<0.0002
Drop >0.1 g/dL (n)	107	48	59	—
0.1-0.49 g/dL (n)	16	3	13	—
0.5-0.99 g/dL (n)	22	14	8	—
1.0-1.49 g/dL (n)	22	14	8	—
1.5-1.99 g/dL (n)	17	7	10	—
2.0-2.49 g/dL (n)	13	5	8	—
2.5-2.99 g/dL (n)	4	3	1	—
>3.0 g/dL (n)	13	2	11	—
Blood transfusions, n (%)	25 (16.3)	11 (14.5)	14 (18.4)	0.52
ESA prescriptions, n (%)	68 (44.4)	31 (40.2)	37 (48.7)	0.331
UTI, n (%)	36 (23.5)	18 (23.4)	18 (23.7)	> 0.999
Time to UTI (days), median (IQR)	22 (9 - 37)	29 (16.75 - 37.75)	17 (8 – 30)	0.20
Time with stent placed (days), median (IQR)	36 (32 - 38)	36 (31 - 38)	36 (34 - 38.5)	0.30
UTIs prior to stent removal, n (%)	22 (61)	11 (61)	11 (61)	
Antibiotics used to treat UTI, n (%)				
Fluoroquinolone	18 (50)	8 (44.4)	10 (55.6)	
Beta Lactams	9 (25)	3 (16.7)	6 (33.3)	
Nitrofuran	3 (8.3)	2 (11.1)	1 (5.6)	
Sulfa	1 (2.8)	1 (5.6)	0 (0)	
IV Antibiotics	5 (13.9)	4 (22.2)	1 (5.6)	
No Treatment	1 (2.8)	1 (5.6)	0 (0)	

Abbreviations: IQR, interquartile range; Hgb, hemoglobin; ESA, erythropoiesis-stimulating agent; UTI, urinary tract infection; Nitrofuran, nitrofuran derivative; IV, intravenous.

### Acute Anemia

**Figure 2.**
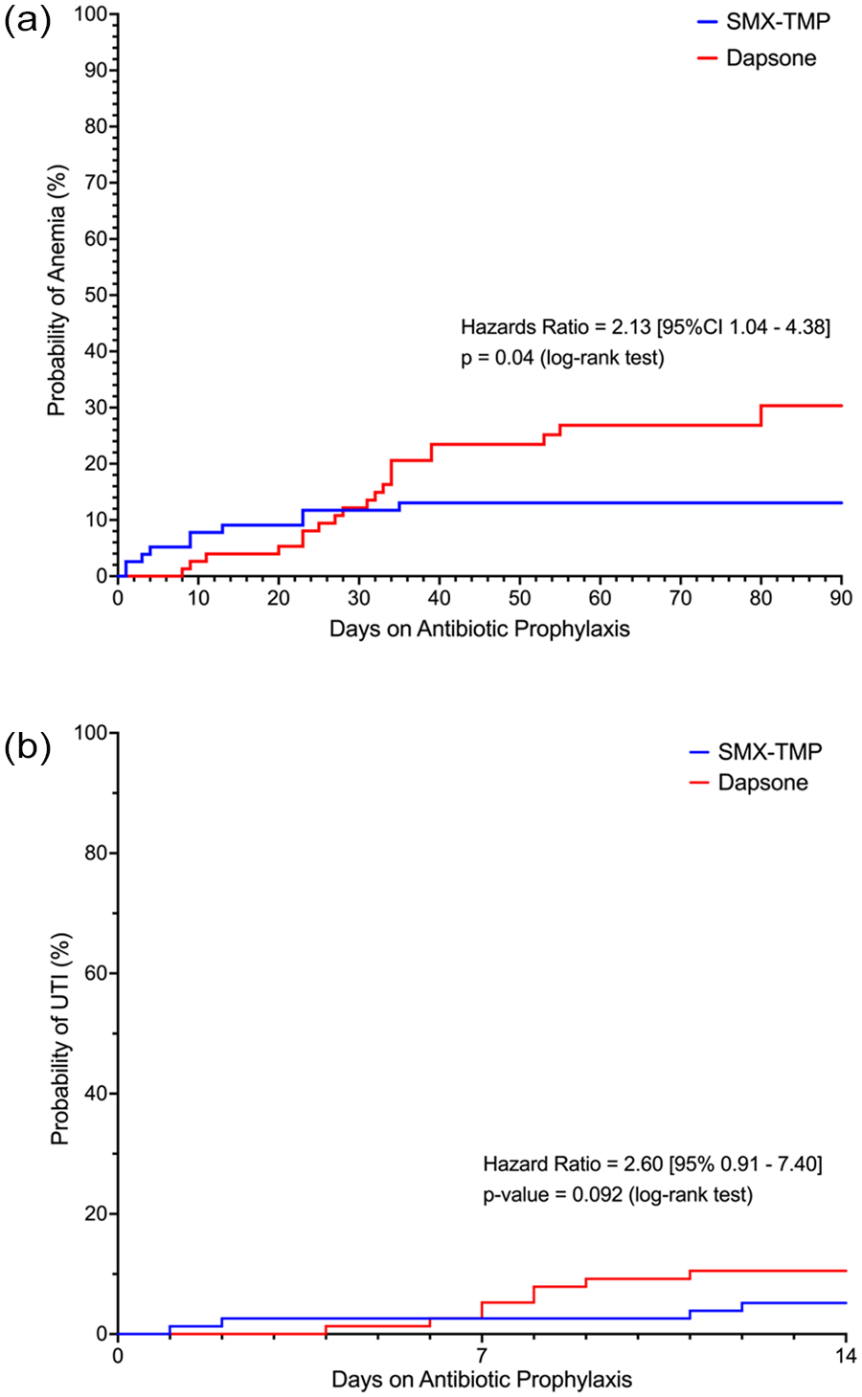
Kaplan-Meier curves showing probability of anemia (a) and UTI on dapsone compared to SMX-TMP (b).

Acute anemia was less common overall (13% vs 26.3%, *P* = 0.043) in patients on SMX-TMP compared with dapsone, respectively (Table 2). For the primary outcome, patients were more likely to experience acute anemia within 90 days of transplant (Figure 2a) while on dapsone (cHR = 2.13, 95% CI = 1.04-4.38, *P* = 0.04).

Dapsone prophylaxis (aHR = 2.27, 95% CI = 1.04-5.30, *P* = 0.047), baseline Hgb (aHR = 1.36, 95% CI = 1.09-1.71, *P* = 0.0069), baseline SCr (aHR = 1.14, 95% CI = 1.02-1.26, *P* = 0.013) were independently associated with an increased risk of acute anemia. The risk of acute anemia was not significantly associated with donor type (aHR = 2.05, 95% CI = 0.86-4.58, *P* = 0.09) or G6PD level (aHR = 0.89, 95% CI = 0.71-1.06, *P* = 0.23). However, a weak, positive correlation was observed between G6PD and Hgb level in patients that received dapsone (*r* = 0.28, *P* = 0.013).

There were no differences in the rates of blood transfusion (14.5% vs 18.4%, *P* = 0.52) or ESA prescriptions (40.2% vs 48.7%, *P* = 0.331, Table 2). The median time to hemoglobin nadir from date of transplant was 6 (IQR = 4-19) and 39 (IQR = 29-63) days for the SMX-TMP and dapsone groups, respectively (*P* < 0.001).

### Urinary Tract Infection

There was no difference in the overall prevalence of UTI at 90 days (23.4% vs 23.7%, *P* > 0.99) between SMX-TMP and dapsone groups, respectively. Urinary tract infection tended to occur earlier in patients in the dapsone group (17 [IQR = 8-30] vs 29 [16.75-37.75] days, *P* = 0.20). There was no difference in UTI risk at any time within the first 14 days after initiation of SMX-TMP or transition dapsone (cHR = 2.46; 95% CI = 0.86-7.04, *P* = 0.092) (Figure 2b). Female sex was the only characteristic associated with increased risk of UTI (aHR = 6.36; 95% CI = 1.87-28.88, *P* = 0.0059) in Cox regression analysis. The risk of UTI associated with dapsone exposure did not achieve statistical significance (aHR = 2.73; 95% CI = 0.85–10.31, *P* = 0.10).

Ureteral stents were maintained for a median of 36 days in both the SMX-TMP (IQR = 31-38) and dapsone (IQR = 34-38.5) groups (*P* = 0.30, Table 2). Most UTIs (11 of 18 patients) occurred pre-stent removal in both groups. Prophylaxis with dapsone (aHR = 2.65, 95% CI = 0.76-10.36, *P* = 0.13) and duration of ureteral stent (aHR = 1.07, 95% CI = 0.94-1.28, *P* = 0.35) were not significantly associated with UTI risk between groups. Intravenous antibiotics were used to treat 3 patients in the SMX-TMP group compared with 1 patient in the dapsone group. Overall, there was a low prevalence of multiple UTIs (1.3% vs 3.9%, *P* = 0.37). Most UTIs were treated with fluroquinolones in both groups (50%).

## Discussion

### Key Findings

This study contributes to the current understanding of anemia and UTI in patients that received prophylaxis for opportunistic infection with daily dapsone and SMX-TMP after renal transplant. Kidney transplant recipients who received dapsone experienced a twofold higher prevalence of acute anemia after controlling for baseline SCr and Hgb, despite G6PD levels within normal limits. Acute anemia appeared to be delayed in patients that received dapsone, which is consistent with the mechanism of the adverse effect. In contrast, acute anemia in the SMX-TMP group developed in close temporal proximity to kidney transplant, suggesting that surgical blood loss likely contributed.

Glucose-6-phosphate-dehydrogenase sufficiency may have attenuated the effect of dapsone on the risk of acute anemia (ie, 2 g/dL decrease). However, the positive correlation observed between G6PD level and any amount of Hgb decline, appears to substantiate the risk of anemia even in patients with G6PD levels that were within normal limits. The shorter duration of prophylaxis with dapsone was likely due to discontinuation to mitigate anemia. However, remarkably, anemia was not associated with an increase in consequent transfusion or ESA prescription. These findings suggest that the impact of dapsone on Hgb levels was relatively moderate for most patients and affirm that transfusion and prescribing practices were reasonably disciplined.

The risk of UTI appeared to be concentrated in close proximity to ureteral stent placement, which is a primary risk factor.^9^ Notably, the overall prevalence of UTI was comparable with similarity between groups over the 3-month period, indicating that the secondary prophylactic effect was nominal for both agents. For SMX-TMP, this finding may be attributed to the relatively low dose (400-80 mg), compromised renal elimination, prolonged ureteral stent exposure, and immunosuppression.

### Implications

The rate of acute anemia (26.3%) observed with KTRs in this study was consistent with previous reports in patients with HIV (23%) and in recipients of heart (22%) and lung (22.7%) transplant who received prophylaxis with dapsone.^5,7,8^ Hedvat et al explored the effect of dapsone (100 mg/d) versus atovaquone on hemoglobin levels in KTRs with normal G6PD levels for 90 days. The median decrease in hemoglobin from baseline in dapsone group was similar to this study (1.3 g/dL vs 0.95 g/dL).^9^ Schumacher et al^10^ compared once-weekly dapsone with SMX-TMP in KTRs and observed a statistically significant median decrease in hemoglobin of 0.20 g/dL in the dapsone group. The higher magnitude of change in hemoglobin observed in this study is likely due to the higher dapsone dose, that is, 100 mg/d compared with 100 mg/wk. This may further support a dose-dependent effect and suggest dose adjustment could mitigate this complication. Neither study evaluated concurrent risk factors for anemia or prevalence of UTI.

In addition to ensuring against G6PD deficiency, the median weight-based dose of dapsone (1.2 mg/kg/d) in this study was well below the limit (≥1.5 mg/kg/day) commonly associated with hemolysis and anemia. Therefore, following the commonly cited dose and G6PD thresholds may not fully protect patients from developing acute anemia. Careful monitoring and risk assessment is necessary to mitigate the risk of this adverse effect.

Reported prevalence of UTI in KTRs in literature ranges from 10% to 98%.^11^ A meta-analysis of 3,364 KTRs showed UTI in 38%.^12^ The rate of UTI observed in this study was lower than reported (35.2%) in a similar single-center study.^13^ This disparity may be attributed to different definitions of UTI diagnostic criteria or length of follow up period. Finally, the risk of UTI coincided with ureteral stent exposure in first 6 weeks post-transplant, supporting previous findings that earlier removal is a protective factor.^14^

### Strengths and Limitations

The study sample was representative of a diverse, real-world population. The study was relatively small; however, the sample size was adequate for the primary outcome of acute anemia. Of note, a much larger sample size (ie, 400 subjects per group) would be necessary to detect a statistically significant difference based on the rates of UTI observed (ie, 10.5% vs 5.2%) in the first 14 days of therapy. It was important to consider the study findings in the context of evolving clinical practice. In this case, the agents and methods employed for prophylaxis of opportunistic pathogens (eg, first-line and second-line agents, duration of prophylaxis) during the study period have not substantially changed. Moreover, practice changes that may have indirectly affected the study outcomes (eg, ESA use, blood transfusion) were unlikely to have evolved significantly in the interim period based on clinical practice guidelines.

The study findings were limited by the retrospective design. However, a prospective study would contend with randomizing patients to an unfavorable, second-line therapy for investigational purposes. Patients that received dapsone were commonly transitioned from SMX-TMP which typically occurs in practice. As a result, they likely benefited from a shorter interval of dapsone exposure and SMX-TMP prophylaxis in the immediate postoperative period. Thus, the expected risks of acute anemia and UTI may be even higher in patients initiated on prophylaxis with dapsone therapy. However, there was no difference in the risk of UTI between SMX-TMP and dapsone groups after controlling for day of prophylaxis initiation and concurrent stent exposure.

Limitations of the single-center design included external validity of the patient population and transplant protocol. The transplant induction therapy used may differ from protocols employed by other centers. Furthermore, induction therapy with ATG was based on actual body weight. Thus, patients in this study may have experienced a higher level of immunosuppression than centers that use ideal or adjusted body weights for ATG dosing. However, this practice was consistent between groups. The effects of other induction agents (eg, basiliximab) on the risk of UTI were beyond the scope of this study as all patients received leukoreduction with ATG.

The definition of acute anemia was defined by a ≥ 2 g/dL decrease, used in a similar study.^6^ A more conservative or liberal cutoff may result in higher or lower rates of anemia. Furthermore, conventional parameters for anemia are less useful in this population as most patients with chronic kidney disease would meet the threshold at baseline. For example, most patients in this study were anemic at baseline with mean Hgb 10 ± 1.6 (Table 1).

Erythropoiesis-stimulating agent exposure was inferred from prescriptions and may not reflect actual use due to variance in access (eg, prior authorization), adherence (ie, self-administration), and utilization (eg, contingency-based). In addition, blood transfusions were subject to similar subjectivity and variability in indication and timing. However, these factors and outcomes were comparable between cohorts. Finally, appropriateness of UTI treatments and resistance patterns were not collected. Therefore, the impact of prophylactic agents on UTI treatment could not be further explored.

### Future Directions

The effect of subtherapeutic doses of SMX-TMP on the risk of drug-resistant infection (eg, UTI) may merit exploration in this population. In addition, characterization of additional risk factors for acute anemia associated with dapsone (eg, glutathione levels) may be necessary to better understand this phenomenon. Finally, further research is necessary to clarify the safety and efficacy of alternative dosing of SMX-TMP (eg, 3 times weekly) to mitigate the risk of hyperkalemia.

## Conclusion and Relevance

The risk of anemia was 2-fold higher in KTRs who received dapsone compared with SMX-TMP but did not appear to affect the rates of ESA use or blood transfusion. There was no difference in the risk of UTI between groups. These findings may help further inform agent selection and monitoring of prophylaxis for opportunistic infection in KTRs.
